# Triglyceride-rich lipoprotein remnants, low-density lipoproteins, and their relative contribution to risk of atherosclerotic cardiovascular disease in the UK Biobank population

**DOI:** 10.1093/eurheartj/ehad337

**Published:** 2023-06-26

**Authors:** Elias Björnson, Martin Adiels, Marja-Riitta Taskinen, Stephen Burgess, Aidin Rawshani, Jan Borén, Chris J Packard

**Affiliations:** 1 Department of Molecular and Clinical Medicine, University of Gothenburg, Gothenburg, Sweden; 2 School of Public Health and Community Medicine, Institute of Medicine, University of Gothenburg, Gothenburg, Sweden; 3 Research Program for Clinical and Molecular Metabolism, University of Helsinki; University of Helsinki, Finland; 4 MRC Biostatistics Unit, University of Cambridge, Cambridge, UK; 5 Cardiovascular Epidemiology Unit, Department of Public Health and Primary Care, University of Cambridge, Cambridge, UK; 6 Institute of Cardiovascular and Medical Sciences, University of Glasgow, Glasgow, UK

**Keywords:** Apolipoprotein B, Mendelian randomisation, remnants, cardiovascular disease, genetics, single-nucleotide polymorphisms, LDL cholesterol, triglyceride

## Abstract

**Background and Aims:**

The gradient of the relationship of triglyceride-rich lipoproteins (TRL) with risk of atherosclerotic cardiovascular disease (ASCVD) compared to low-density lipoprotein (LDL) is yet to be resolved fully.

**Methods and Results:**

Subjects were 354,104 UK Biobank participants not on lipid-lowering treatment in whom TRL/remnant-cholesterol(-C) was estimated. Single nucleotide polymorphisms (SNPs) associated with TRL/remnant-C and LDL-C were identified and the relationships between SNP effects on lipids, apoB, and ASCVD explored using multivariable Mendelian randomisation. LDL-C and TRL/remnant-C associated independently with ASCVD with respective odds ratios (ORs) per 1 mmol/L increase of 1.24[95%CI:1.15-1.32] and 1.93[95% CI:1.51-2.47]. TRL/remnant-C and apoB were independently related to ASCVD risk with OR per standard deviation (SD) of 1.20[95%CI:1.11-1.29], and 1.19[95%CI:1.13-1.26] respectively. Two SNP clusters were identified based on effects on TRL/remnant-C relative to apoB. Cluster 1 which included SNPs associated with genes likely to influence receptor-mediated pathways, had effects on TRL/remnant-C, LDL-C and apoB whereas cluster 2, which included SNPs associated with genes likely to affect lipolysis, impacted mainly TRL/remnant-C and plasma triglyceride. The gradient of apoB with ASCVD risk in cluster 2 (OR=1.61[95%CI:1.46-1.78] per SD change) was greater than in cluster 1 (OR=1.31[95%CI:1.26-1.36] per SD change). A concordant result was obtained by constructing gene scores for each cluster; hazard ratios per 10mg/dL increase in apoB were 1.25[95 % CI:1.16-1.35] for cluster 2 and 1.12[95 %CI:1.09-1.15] for cluster 1.

**Conclusions:**

Distinct SNP clusters appear to impact differentially on remnant metabolic pathways. Our findings are consistent with TRL/remnant particles having a greater atherogenic potential than LDL.

## Graphical abstract

**Graphical Abstract:**
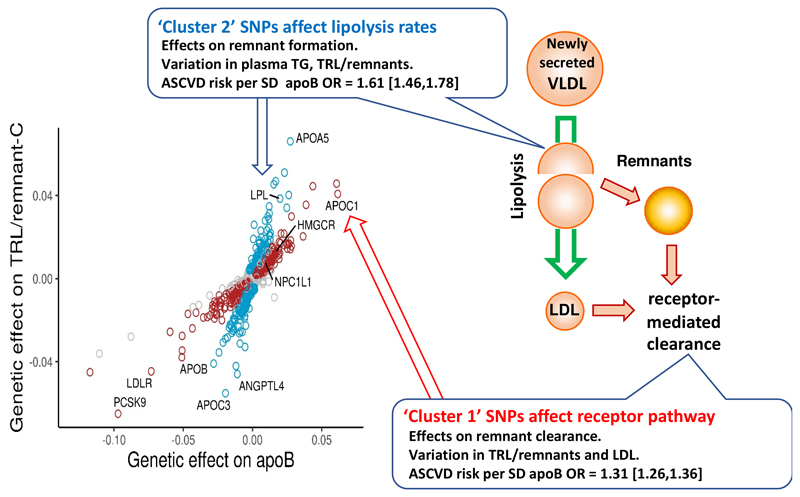

## Introduction

Genetic studies reveal that the association between triglyceride-rich lipoproteins (TRL) and atherosclerotic cardiovascular disease (ASCVD) is likely causal^1, 2^, although the features of these lipoproteins that promote development of atherosclerosis are not yet clear.^2^ Most attention to date has focussed on the cholesterol content of TRL and their ‘remnants’, the products of partial lipolysis of apolipoproteinB48-containing chylomicrons and apoB100-containing very-low density lipoproteins (VLDL).^1–5^ In analogy with the pathogenic mechanisms linked to low-density lipoproteins (LDL), remnant lipoproteins can penetrate the sub-endothelial space in artery walls and bind to proteoglycans, thereby initiating cholesterol deposition and foam cell formation.^2, 6^

Questions arise as to the strength of the relationship of TRL/remnant cholesterol (TRL/remnant-C) relative to the well-understood benchmark of LDL cholesterol (LDL-C)^7^ [note the term ‘TRL/remnant’ is used throughout to recognise the fact that there is no clear definition of remnant particles that allows them to be identified separately from other TRL; they are part of a continuum].^2^ The issue has been addressed in a number of recent reports and differing conclusions have been drawn. On the one hand, the ASCVD risk associated with a unit change in plasma apoB linked to variation in genes known to affect triglyceride (TG) metabolic pathways (e.g. lipoprotein lipase) was shown to be quantitatively similar to the risk of the same change in apoB due to variation in genes affecting LDL metabolism (e.g. the LDL receptor).^8^ This observation led to the concept that the atherogenic potential of TRL and LDL were broadly the same, and risk was a function of the number of apoB-containing particles in the circulation.^9, 10^Other studies have provided evidence that the risk linked to a given increase in TRL/remnant-C - measured or estimated using a range of methodological approaches – is substantially greater than that associated with the same increase in LDL-C.^1, 11–13^ Resolving these discordant findings is important since it impacts on risk assessment, and the design and interpretation of intervention trials.

In the present study, we undertook an evaluation of the relationship of TRL/remnants to ASCVD risk in a large, well-characterised population – the UK Biobank - taking advantage of the fact that an indirect measure of the cholesterol content in TRL/remnants could be derived by subtracting the directly assayed LDL-C concentration from non-high-density lipoprotein (non-HDL-C). We also examined the nature of the genes influencing TRL/remnant-C levels in light of the known metabolic properties of these lipoprotein species.^2^ Our findings support the view that TRL/remnant particles carry a greater atherogenic potential than LDL. Further, it was possible to identify a cluster of SNPs that appeared to affect primarily TG and TRL/remnant-C, and a separate cluster that has a substantial impact on both TRL/remnant-C and LDL-C.

## Methods

### Study population

This investigation utilised the UK Biobank cohort (over 502,000 UK residents of mainly white ancestry).^14^ For the majority of analyses we selected 354,104 subjects who had the required plasma lipid levels recorded and were not on lipid-lowering therapy at baseline (Supplementary Figure 1).

### Lipid measurements

LDL-C was measured directly (Beckman Coulter, Brea, CA) (Data field 30780). Non-HDL cholesterol was determined as the difference between plasma cholesterol (Data field 30690) and HDL cholesterol (Data field 30760).^14, 15^ TRL/remnant-C was derived by subtracting LDL-C from non-HDL-C and since it was based on measured parameters, it was deemed an indirectly ‘measured’ concentration. The cholesterol content of TRL was also estimated from the equation published by Sampson et al^16^ which is based on plasma TG and non-HDL-C concentrations. To distinguish these two variables, we refer to the result from the Sampson equation as ‘very low-density lipoprotein cholesterol – ‘VLDL-C’. All other analytes were measured by standard laboratory methods (see online showcase of UK Biobank methods: https://biobank.ctsu.ox.ac.uk).

### Genetic analyses

Genotyping with the UK BiLEVE Axiom or UK Biobank Axiom arrays provided an evaluation of 805,426 single nucleotide polymorphisms (SNPs) spanning the entire genome (Supplementary Figure 1).

Three approaches were used to investigate the impact of genetic variation on TRL/remnant-C, TG, VLDL-C, LDL-C and apoB as lipoprotein variables, and the associated effect on ASCVD risk.

First, we undertook both a ‘targeted gene analysis’ using a set of SNPs linked to genes known to be involved in lipoprotein metabolism (n=61, listed in Supplementary Table 1) and an analysis using previously published lipid-related SNPs (n=178).^8, 17^

Second, a new genome-wide association study (GWAS) adjusted for age, sex and genetic principal components 1-5 was performed to identify SNPs associated with LDL-C and/or TRL/remnant-C. SNP selection was based on significance thresholds of reducing stringency; for Tier1 SNPs the threshold was <1×10^-21^, for Tier 2 SNPs it was <1×10^-12^, and for Tier 3 SNPs it was <5×10^-8^ (the usual value for nominal GWAS significance). SNP sets were pruned for linkage disequilibrium (r^2^ <0.3) and minor allele frequency (threshold >0.01). If two SNPs were in linkage disequilibrium, the SNP with the largest combined effect size (square root of [LDL-C effect size squared plus TRL/remnant-C effect size squared]) was selected. The list was further filtered for association (Bonferroni-Holms adjusted P<0.05) with lipoprotein(a), and the presence of at least 1000 heterozygous subjects per allele. This process yielded 380 SNPs for Tier 1, 752 SNPs for Tier 2 and 1222 SNPs for Tier 3 (Supplementary Figure 1).

Third, gene scores for subsets of SNPs (in clusters as described below) were created by identifying the apoB raising allele for each SNP as the exposure allele. For each subject a score was generated as the weighted sum of the number of apoB raising alleles present. The cohort was then divided into deciles of gene score and mean levels of apoB, TG, TRL/remnant-C and LDL-C determined.

### ASCVD outcomes

These are defined in Supplementary Table 2. For studies of the association with genetically-determined lipid levels, outcomes were the combination of prevalent and incident events (myocardial infarction [MI] and coronary revascularisation). For studies based on observational data, outcomes were incident events occurring during the approximately 12-year follow up period.

### Statistical methods

All statistical analyses were performed using R version 4.0.4. Multivariable Mendelian randomisation (MR) analyses based on the inverse variance-weighted (IVW) method (which assumes all variants are ‘valid’ instrumental variables; that is the SNP effect on outcome is solely through its effect on the exposure/risk factor^18^) were performed using data from the 354,104 subjects who were not on lipid-lowering therapy at baseline and had all required lipid measurements. The possible impact of pleiotropic effects (SNP variants influencing the outcome out with their effect on the exposure) was examined using the MR-Egger^19^ and the MR ‘contamination mixture’ methods (see Supplementary Tables 3 and 4 for a fuller discussion).^18^ Odds ratios for ASCVD outcomes were determined per unit change (1.0 mmol/L for lipids or 1.0 g/L for apoB) and per population standard deviation (SD) in the variable of interest.

Gene scores were formulated as described above to provide an aggregate assessment of the relationships between genetic variation in apoB and that in TRL/remnant-C, TG, and LDL-C for defined clusters of SNPs. Cox proportional hazards models were used to determine for each decile of the score a hazard ratio for incident ASCVD outcome over the 12-year follow up (Supplementary Table 2). This analysis was performed on 401,771 subjects without a history of MI or coronary revascularization at baseline (an apoB level was essential for inclusion but lipid levels could be missing). For each cluster, an overall hazard ratio for ASCVD risk was scaled per 10 mg/dL increase in apoB.

## Results

The UK Biobank cohort comprises 502,460 men and women 56.5 years old at enrolment of which 415,575 were not on lipid-lowering medication at baseline (selected so as not to have the confounding effect of therapy on relationships between lipid variables). For 354,104 subjects, TRL/remnant-C could be derived by subtracting direct assayed LDL-C from non-HDL-C. TRL/remnant-C correlated strongly with TG (r^2^=0.63, P<0.0001) and moderately with LDL-C (r^2^=0.37, P<0.0001) (Supplementary Figure 2A). The association of TRL/remnant-C with LDL-C was stronger for subjects with plasma TG <4.0 compared to those with higher TG (Supplementary Figure 2B). VLDL-C estimated using the Sampson equation ^16^ exhibited a strong association with TG (r^2^=0.97, P<0.0001)). Overall, TRL/remnant-C correlated with VLDL-C (r^2^=0.74, P<0.0001) (Supplementary Figure 2A) but the strength of the association varied by TG range. TRL/remnant-C and VLDL-C gave similar values for subjects with plasma TG<2.5 mmol/l but in those with TG 2.5-4.0 mmol/l, VLDL-C was higher than TRL/remnant-C by about 0.3 mmol/L, and for TG >4.0 mmol/L the discrepancy was about 0.7 mmol/L (Supplementary Figure 2C).

### Relative risk of an ASCVD event associated with change in TRL/remnant-C *vs*. LDL-C based on genetic and observational data

In multivariable Mendelian randomisation, the odds ratio for an ASCVD outcome per 1.0 mmol/L genetically-defined increase in TRL/remnant-C was greater than that for LDL-C (Table 1). This was true whether the SNPs were linked to targeted genes (as listed Supplementary Table 1), the previously published SNP set, or the tiered SNP sets from the GWAS; similar point estimates were observed in all models. It was noteworthy (i) that the odds ratio for LDL-C in univariable analysis at 1.49 [95%CI 1.39-1.59] was reduced to 1.28 [95%CI 1.18-1.39] when TRL/remnant-C was included in the model (the targeted gene analysis univariable result is given in Table 1, and Supplementary Table 3 gives results with other SNP sets), and (ii) that both lipid variables were independent risk predictors in multivariable models. The possibility that SNP pleiotropic effects had biased the results was subject to detailed statistical evaluation and found to be unlikely (Supplementary Table 3). Supplementary Figure 3 provides analogous models for VLDL-C but here, in contrast to TRL/remnant-C, it can be seen that the odds ratios per 1.0 mmol/L genetically-determined increase were not different from those for LDL-C.

In Cox proportional hazard models based on observational data, per 1.0 mmol/L increase the hazard ratio for LDL-C was 1.20 [95%CI:1.16-1.24] compared to 1.51 [95%CI:1.41-1.63] for TRL/remnant-C (Supplementary Figure 4A). Again, when VLDL-C replaced TRL/remnant-C in the model the results differed in that the hazard ratio at 1.30 [95%CI:1.24,1.36] was closer to that of LDL-C (Supplementary Figure 4B).

### Independence of associations of TRL/remnant-C, VLDL-C, and apoB with ASCVD outcome

Multivariable Mendelian randomisation was used to test the association of lipid variables with ASCVD outcome in models that included apoB (using either the set of 178 previously published SNPs or the 1222 Tier 3 SNPs) (Table 2). We found that TG was a significant independent predictor alongside apoB (Models 1, 2). When TRL/remnant-C was included with apoB, both were significant predictors (Model 3) as was the case also for VLDL-C (Model 4). Using the Mendelian randomisation-Egger method as a sensitivity analysis (Supplementary Table 4), it was found that for the SNPs identified by GWAS results were essentially the same as with the inverse-variance weighted method (Table 1). However, for the 178 SNP set, the significance for TG in a model with apoB was borderline at P=0.026 (Supplementary Table 4).

**Figure 1:**
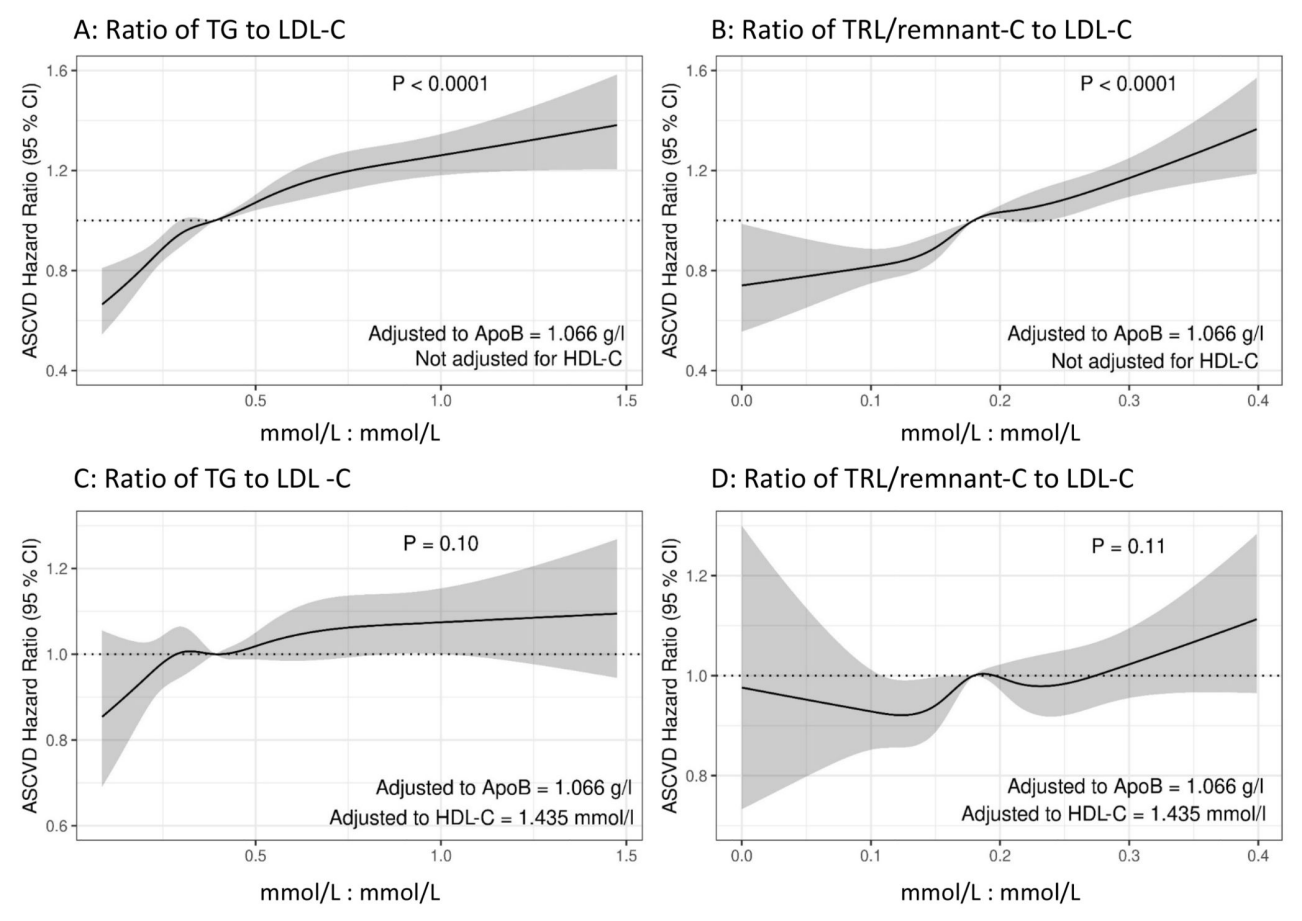
Impact of relative abundance of TG or TRL/remnant-C versus LDL-C on risk of ASCVD event. In statistical models controlling for plasma apoB the influence of variation in the ratio of TG or TRL/remnant-C to LDL-C on the hazard ratio for an incident ASCVD event (MI + coronary revascularisation) was determined. Models were adjusted to apoB=1.066 g/L, sex=female, BMI=26.32 kg/m^2^, age =56 years, systolic BP=137 mmHg, HbA1C=34.8 mmol/mol. In panels A and B, HDL-C was not included in the model while in panels C and D, the models were further adjusted to HDL-C =1.435 mmol/L. P-values of the respective term (TG per LDL-C and TRL/remnant-C per LDL-C) in the Cox proportional hazards models are indicated in each panel.

In an analysis using observational data (reportedly recently using the same data set^10^) the ratio of baseline TG to LDL-C was plotted against incident ASCVD outcome while controlling for apoB concentration (Figure 1 A, C) and the same plot was generated for the ratio of TRL/remnant-C to LDL-C (Figure 1 B, D). In each case, two spline fit models were constructed, one excluding HDL-C as a covariate (**Panels A, B**) while the other adjusted for HDL-C (**Panels C, D**). In the upper panels as the ratio of TG or TRL/remnant-C rose relative to LDL-C there was a substantial and significant increase in ASCVD risk (P<0.0001 in both cases). When HDL-C was included in the model, the positive association was blunted and became non-significant.

### Genetically determined relationship of TRL/remnant-C to apoB

**Figure 2:**
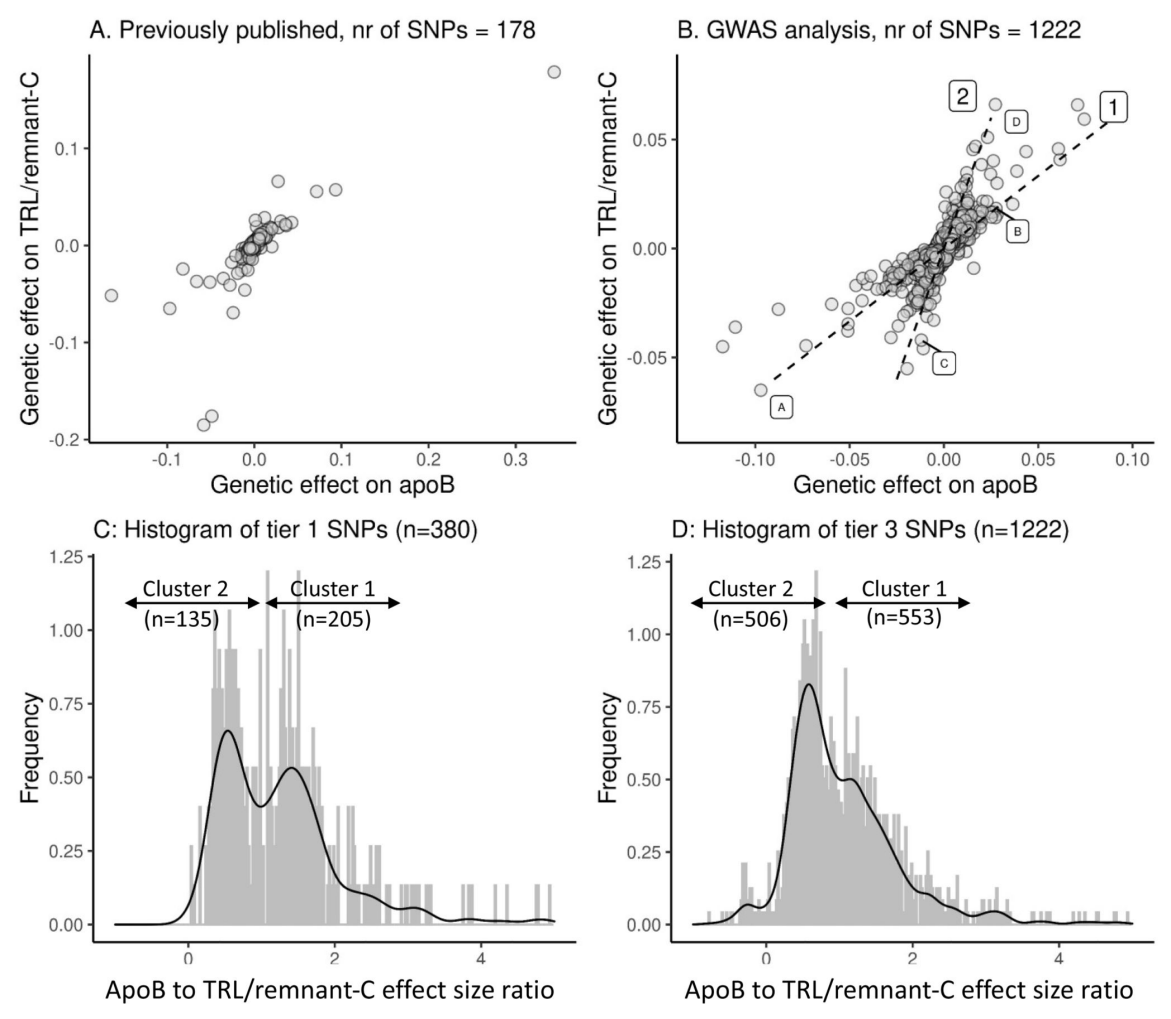
Identification of genes influencing TRL/remnant cholesterol. SNPs were selected from a previously published SNP set (Panel A) or identified from a new GWAS (Panel B). Tier 3 SNPs shown in Panel B were associated with TRL/remnant-C and/or LDL-C at a significance threshold of <5x10^-8^. Effects are expressed as minor allele/major allele. Annotations ‘1’ and ‘2’ in Panel B denote putative SNP clusters defined by the gradient of effect size on apoB relative to effect size on TRL/remnant-C. Units for apoB are g/L and for TRL/remnant-C mmol/L. For example, SNPs rs11591147 in *PCSK9* (denoted ‘A’) and rs1367117 in *APOB* (‘B’) had effect size ratios of 1.49 and 1.50 while SNPs rs328 in *LPL* (‘C’) and rs3135506 in *APOA5* (‘D’) had ratios of 0.29 and 0.41 respectively. Panels C and D give the frequency distributions for values of the ratio of apoB effect size to TRL/remnant-C effect size (beta coefficients ratio) for each SNP in the Tier 1 and Tier 3 sets respectively. The arrows denote the values used to divide the SNPs in to cluster 1 (0.9 to 3.0) and cluster 2 (-0.75 to +0.75). Numbers of SNPs allocated to each cluster are shown. The number of unallocated SNPs (not falling into the denoted intervals) was 40 for Tier 1 and 163 for Tier 3.

In previously published studies, the lipid variables used to assess the relationship of TRL/remnant particles to ASCVD risk were primarily total apoB and the cholesterol content of TRL. ^1, 8^ Prior to evaluating their association with ASCVD risk, we sought to understand better the quantitative nature of the relationship between these two variables, and the influence of genes that affect their concentrations. More specifically, we addressed the question as to whether all genetic variants that cause concomitant changes in both variables generate the same magnitude of change (effect size) in TRL/remnant-C for a given change in apoB. Two approaches were adopted. In the first, we explored the impact of variation in published candidate genes affecting lipid metabolism.^8^ In the second we used the GWAS Tier 3 SNP set, a broad range of variants influencing TRL/remnant-C and /or LDL-C (Supplementary Figure 1). The results are shown in Figure 2. Note, in order not to define an exposure allele in these exploratory investigations (since we wished to examine the interrelationships of multiple lipid-related variables), results are expressed as the effect (sign and magnitude) of the minor allele relative to the major allele.

Both analyses yielded similar patterns in that variation in TRL/remnant-C was accompanied by change in apoB (Figure 2 A, B). For the previously published SNP set (Figure 2A) it can be seen that SNPs increased or decreased both apoB and TRL/remnant-C concomitantly along a broadly uniform gradient, although there was a degree of scatter for SNPs having the largest effect. Examining the larger number of Tier 3 SNPs, it appeared that the degree of ‘scatter’ increased, especially again for those SNPs causing the greatest changes (Figure 2B). This pattern in the data prompted exploration of an alternative view, that the SNPs fell into two main groups defined by differing gradients of association of apoB to TRL/remnant-C (as indicated by the dotted lines labelled‘1’ and ‘2’ in Figure 2B). This view was reinforced by inspection of the frequency distribution of the apoB to TRL/remnant-C effect size ratios which indicated the presence of a bimodal distribution of values (Figure 2 C, D) whether SNPs had been identified using a stringent (Tier 1) or nominal (Tier 3) GWAS significance threshold.

### Definition of SNP clusters

The observations in Figure 2 were explored further by assigning each SNP to more formally defined clusters. SNPs having an effect size ratio (change in apoB relative to change in TRL/remnant-C for the minor compared to major allele) between 0.9 and 3.0 were assigned to cluster 1: SNPs having an effect size ratio of -0.75 to +0.75 were assigned to cluster 2 (Figure 2C, D). Supplementary Table 5A-C illustrates the outcome of this categorisation by giving the effect sizes and apoB to TRL/remnant-C ratio for the 40 SNPs with largest effect size allocated to clusters 1 and 2.

### Relation of apoB to lipid variables in SNP clusters

**Figure 3:**
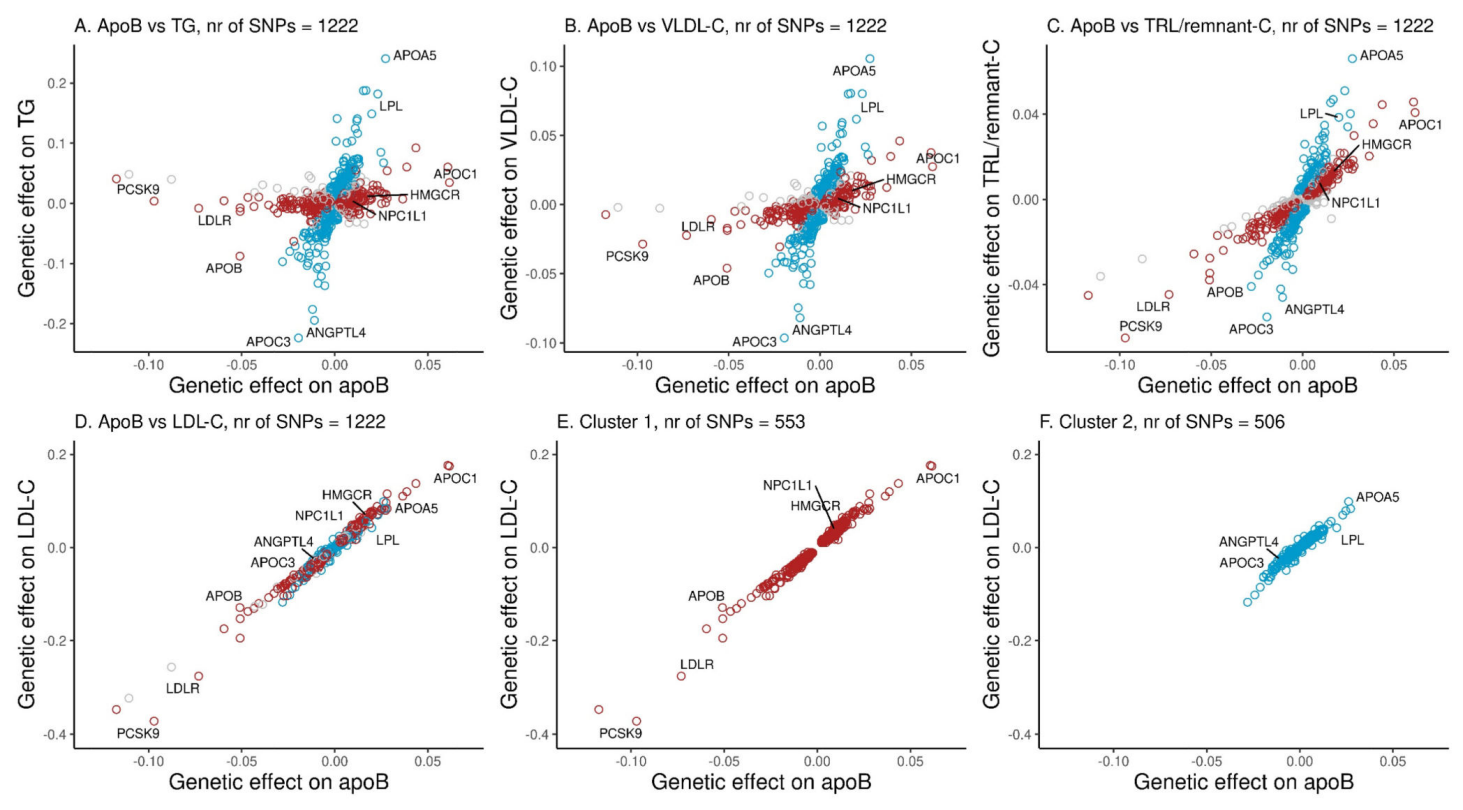
Influence of SNP clusters on plasma TG, TRL/remnant-C, VLDL-C and LDL-C relative to change in apoB. SNPs in the Tier 3 set were allocated to clusters according to the ratio of the effect on apoB relative to the effect on TRL/remnant-C. SNPs assigned to cluster 1 (n=553) are denoted by red circles; those assigned to cluster 2 (n=506) are denoted by blue circles. Unassigned SNPs (n=163) are indicated in grey. Effects are expressed as minor allele/major allele. Units for apoB are g/L and for TG, TRL/remnant-C, VLDL-C and LDL-C are mmol/L. SNPs in each cluster with the largest effect size for TRL/remnant-C, and their effect size ratios (apoB relative to TRL/remnant-C) are given in Supplementary Table 5.

The relationship of apoB to plasma TG, VLDL-C, TRL/remnant-C and LDL-C for the cluster 1 versus cluster 2 variants (using the Tier 3 SNP set) is presented in Figure 3. When the SNP clusters were examined in a plot of TG vs apoB, the distinction between the two was even more pronounced. Cluster 1 SNPs (n=553) had little impact on TG while cluster 2 SNPs (n=506) showed a marked influence on TG (Figure 3, **Panel A**). Cluster 1 SNPs, however, did influence the cholesterol content of TRL with the effect being more marked for TRL/remnant-C (**Panel C**) than for VLDL-C (**Panel B**). For cluster 2 SNPs, a relatively steep association of TG, VLDL-C and TRL/remnant-C with apoB was consistently present. In contrast, the gradient of change in LDL-C relative to change in apoB appeared uniform in both clusters (Figure 3, **Panels D-F**). It was noteworthy, however, that cluster 1 SNPs gave a broad range of effect size for LDL-C (Figure 3E) whereas for cluster 2 SNPs, the range was narrower but still evident (Figure 3F).

Our interpretation of the data presented in Figures 2 and 3 is that the genetic loci influencing TRL/remnant-C apparently fell into two broad categories. SNPs in cluster 1 influence strongly both TRL/remnant (as reflected in TRL-remnant-C) and LDL (as reflected in LDL-C) particle concentrations, and both effects contributed to the variation in plasma apoB. Examples of SNPs in this cluster that have the largest effect sizes include variants in the genes for proprotein convertase subtilisin/kexin9 (PCSK9), the LDL receptor, apoE and apoB (as annotated in Figure 3, see also Supplementary Table 5A). While cluster 1 SNPs had only a modest influence on TG, cluster 2 SNPs, in contrast, exhibited a strong influence on TG and TRL/remnant-C, and had more moderate effects on LDL-C and apoB (Figure 3). Examples of SNPs in cluster 2 (as annotated in Figure 3, see also Supplementary Table 5B) include variants in the genes for apoA5, apoCIII and lipoprotein lipase. Importantly, the division into clusters provided SNP sets that although they affected genetically-defined TRL/remnant-C concentrations to a similar degree (Figure 3C) differed markedly (about 3-fold) in their effects on TRL/remnant-C vs LDL-C (Supplementary Figure 5)

### Association of apoB with ASCVD risk in separate SNP clusters

**Figure 4:**
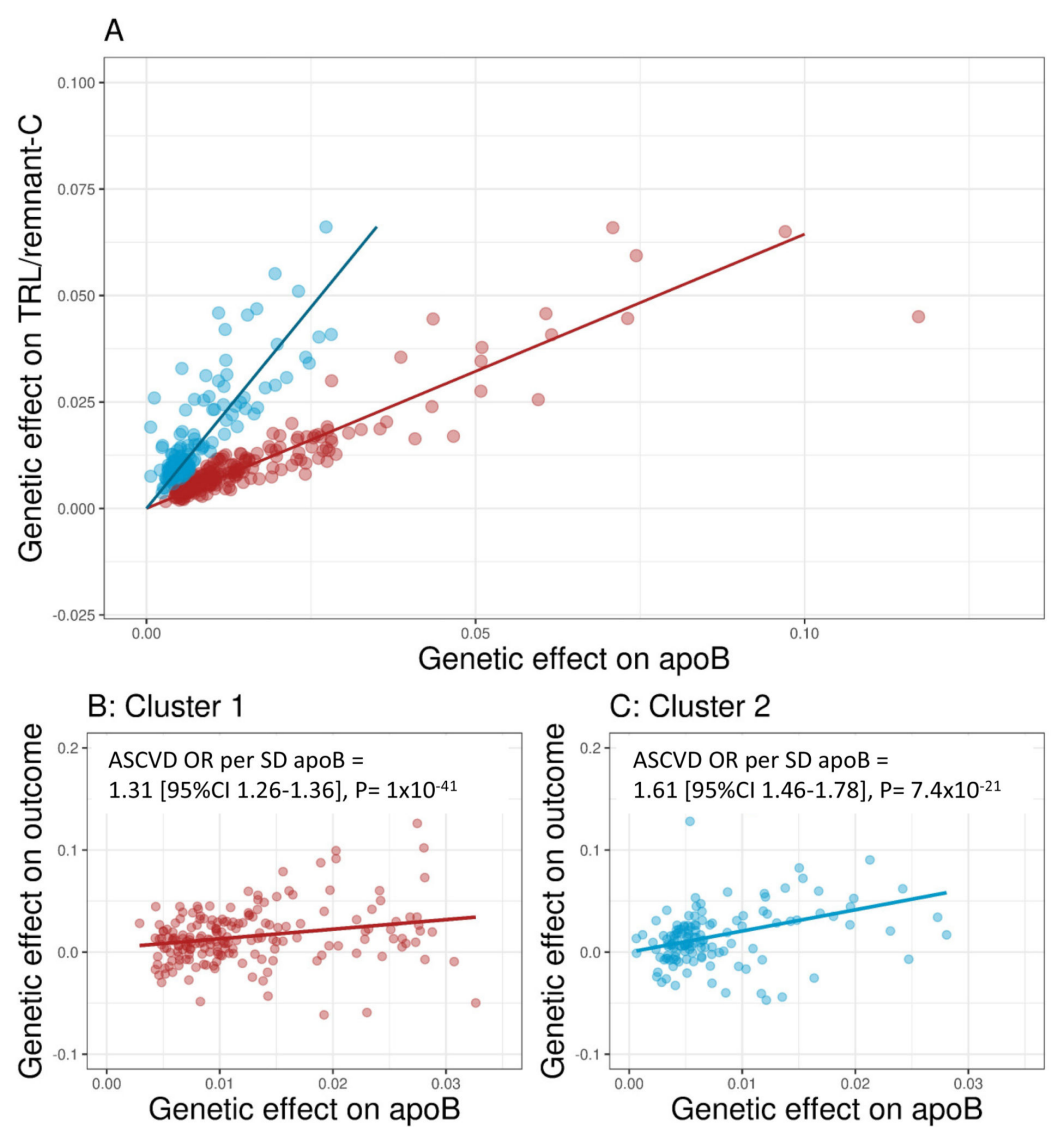
Association of apoB with TRL-C and ASCVD risk in clusters 1 and 2. Panel A shows for Tier 1 SNPs the association of TRL-C with apoB in each cluster with the exposure allele defined as the variant raising apoB. As for the Tier 3 set in Figure 3, the 380 Tier 1 SNPs were assigned to clusters on the basis of the ratio of effects on apoB relative to TRL/remnant-C; 205 SNPs were allocated to cluster 1, and 135 to cluster 2. Panels B and C show for cluster 1 and 2 respectively each SNPs’ effect on apoB and on ASCVD (prevalent + incident) outcome (note that the x-axis for cluster 1 in Panel B has been truncated to allow better visual comparison with the apoB range for cluster 2 in Panel C). Mendelian randomisation modelling (inverse-variance weighted method) was used to determine for each cluster the odds ratio per population SD change in apoB (0.23 g/L). The ASCVD outcome effect estimates per SD apoB differed for cluster 1 and cluster 2 with non-overlapping 95%CIs as shown. Results were similar for Mendelian randomisation analysis using the contamination mixture method which accommodates potential pleiotropic effects; odds ratio per SD apoB were for cluster 1 SNPs 1.32 [95%CI 1.26-1.42] and for cluster 2 SNPs 1.65 [1.47-1.90] (Supplementary Table 3). The genetic effect on apoB has units of g/L and on TRL/remnant-C (Panel A) has units of mmol/L.

To assess the association of apoB with ASCVD risk in each cluster, the exposure allele was defined as the variant that raised apoB. **Panels A in** Figure 4 and Supplementary Figure 5 give the gradient of association of TRL/remnant-C with apoB for each SNP cluster using stringent Tier 1 SNPs (Figure 4) or the broader range of Tier 3 SNPs (Supplementary figure 6). **Panels B and C in** Figure 4 **and Panels B-D in** Supplementary Figure 6 show for each cluster the relationship of genetically-determined increase in apoB to risk of an ASCVD event. For the Tier 1 SNP set the odds ratio for an ASCVD outcome per population SD change in apoB was 1.31 [95%CI:1.26-1.36] in cluster 1 versus 1.61 [95%CI:1.46-1.78] in cluster 2 (Figure 3 B, C), and similar results were obtained for clusters from the Tier 3 SNPs (Supplementary Figure 6). As before, the possible impact of SNP pleiotropic effects was subject to statistical evaluation and the calculated ASCVD odds ratios for change in apoB in the clusters appeared unaffected by potential pleiotropic bias (Supplementary Table 3).

Adopting a similar presentation for the targeted gene set (Supplementary Table 1) revealed that the same difference in gradient of genetically-determined apoB with ASCVD outcome was evident for lipolysis-related genes (*LPL*, *APOC3)* versus receptor-related genes (*LDLR*, *PCSK9*) (Supplementary Figure 7).

**Figure 5:**
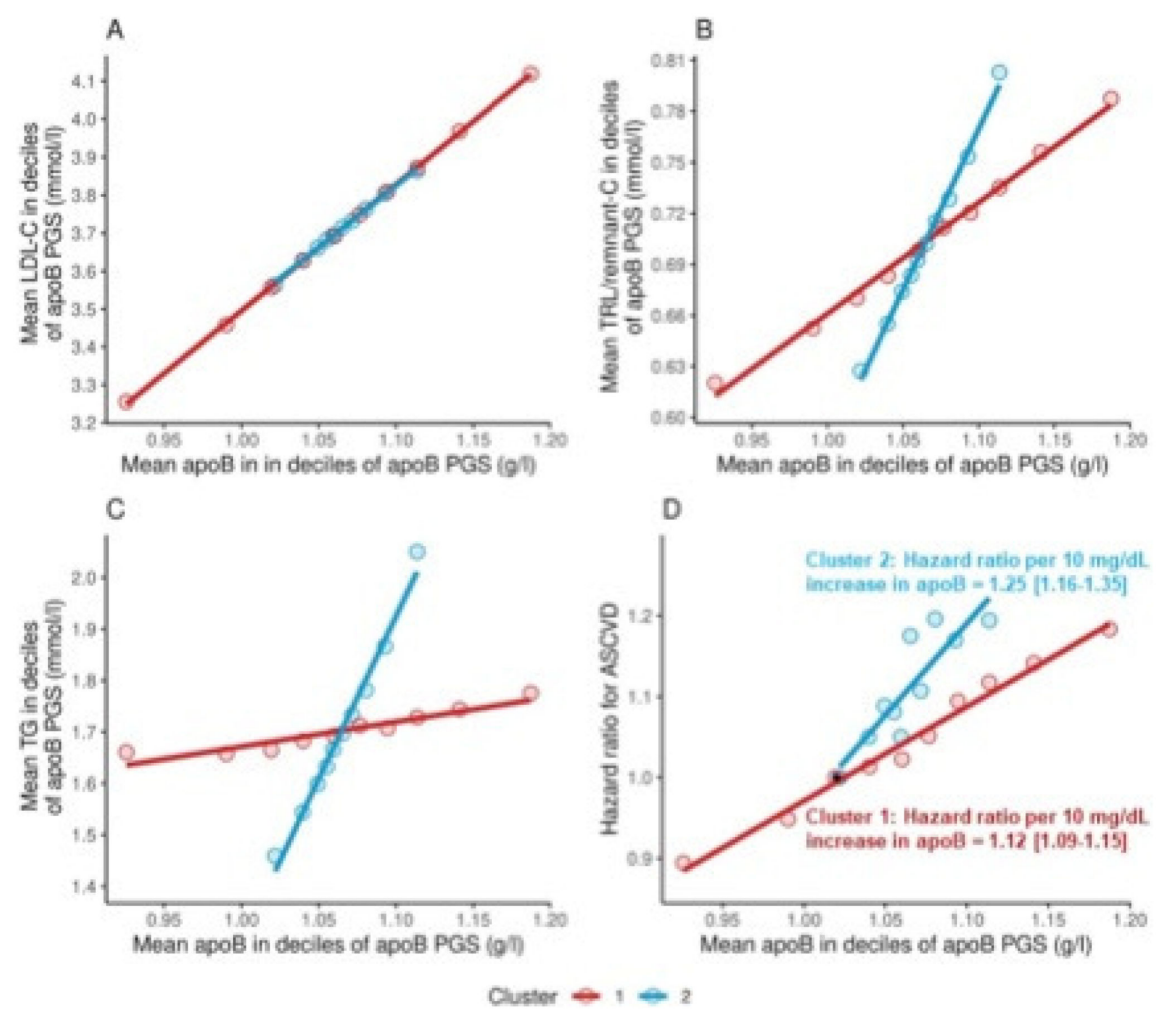
Association of polygenic scores for cluster 1 and 2 with LDL-C, TRL/remnant-C, TG and ASCVD risk. Polygenic scores (PGS) were constructed for each subject summating the apoB raising alleles (multiplied by their beta coefficient) present in cluster 1 and cluster 2 taken from the GWAS Tier 3 SNP set. The cohort was divided into deciles of PGS and mean measured apoB, LDL-C, TRL-C and TG levels at baseline were determined (Panels A to C). The hazard ratio for ASCVD events (incident MI + coronary revascularisation over the 12 year follow up) was estimated for each decile of PGS in each cluster by Cox proportional-hazards modelling. The black dot in panel D denotes the common reference point of 1.02 g/L apoB. For each cluster, hazard ratios were determined also per 10mg/dL increase in apoB as shown in Panel D.

Constructing gene scores based on the two clusters from Tier 3 SNPs gave an indication of the aggregate effects of these variants on plasma lipid levels and ASCVD risk (Figure 5). In these analyses conducted on subjects free of ASCVD at baseline, observed apoB was related to LDL-C, TG, TRL/remnant-C at baseline, and to incident ASCVD events during follow-up. The gene scores for clusters 1 and 2 showed superimposable associations of apoB to LDL-C (**Panel A**) and a clear differentiation with respect to change in apoB and change in TRL/remnant-C, and TG across the deciles of the scores (**Panels B, C**). A difference in gradient between clusters in the quantitative relationship of apoB to risk of an ASCVD event was again evident (**Panel D**). Per 10mg/dL increment in apoB, the hazard ratio for cluster 2 (1.25 [95 % CI: 1.16-1.35]) was significantly greater than that for cluster 1 (1.12 [95 % CI: 1.09-1.15]). Also, in a regression analysis the interaction term ‘apoB x cluster’ was significant at P=0.007 indicating a difference in slope between clusters 1 and 2. In a combined model in the whole cohort with both polygenic scores included, the hazard ratios were 1.12 [95 %CI: 1.09-1.15] and 1.23 [95 % CI: 1.15-1.33] for clusters 1 and 2 respectively.

## Discussion

**Figure 6:**
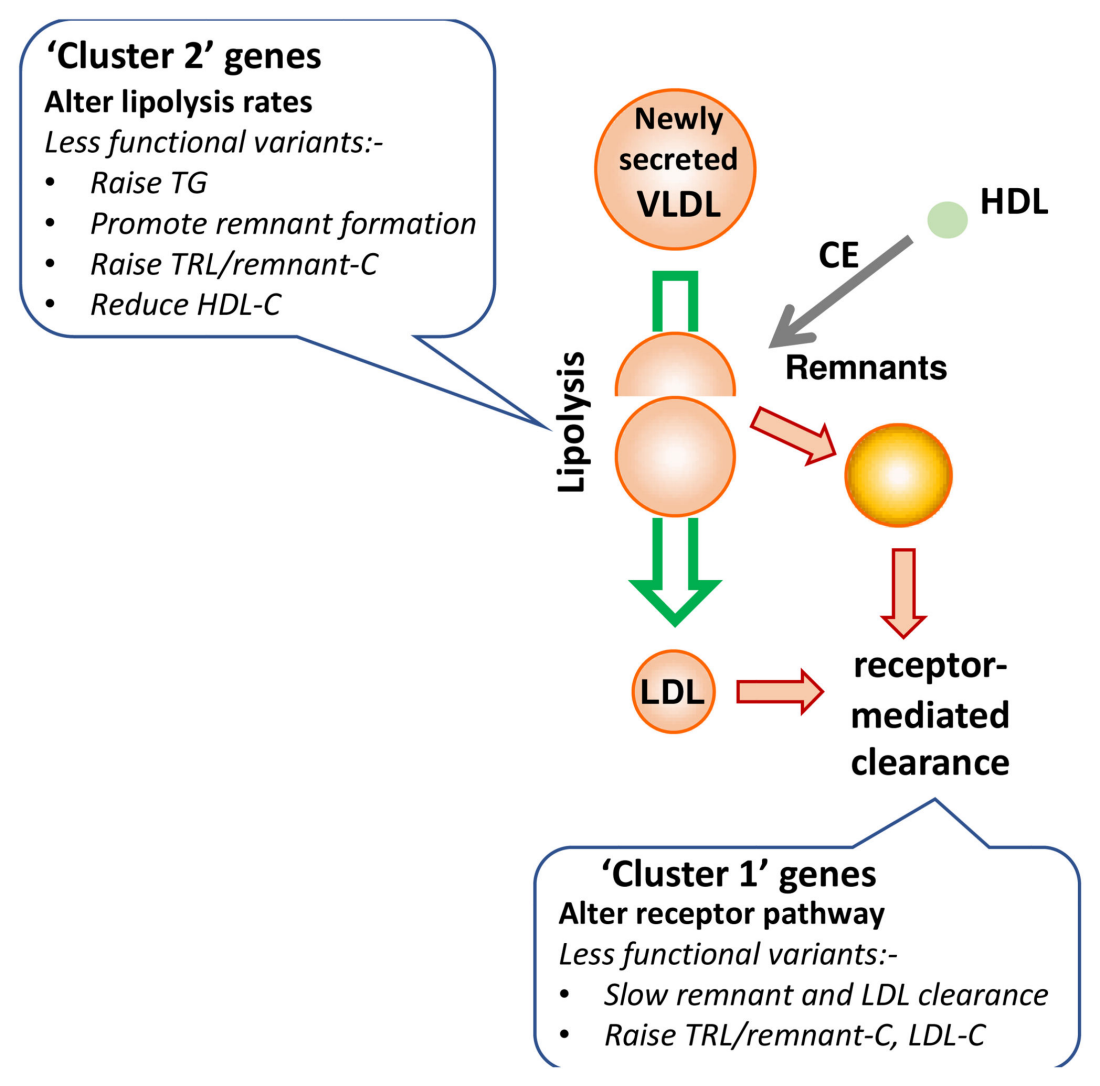
Impact of variation in genes influencing lipolysis and lipoprotein receptors on TRL/remnant and LDL metabolism. In this schematic the putative differential effects of variants of reduced functionality are depicted in boxes. Remnants are defined as lipoprotein particles that have undergone partial lipolysis and remodelling with removal of some core TG and the acquisition of cholesteryl ester by cholesteryl ester transfer protein – mediated exchange.^2, 20, 29^ SNPs in genes that cause a reduction in the efficiency of lipolysis (‘cluster 2’ SNPs) potentially increase the rate of TRL/remnant particle formation but have smaller effects on LDL. Since TRL/remnants contain apoB there is a modest increment in plasma total apoB levels. Further, when lipolysis is slowed there is increased opportunity for cholesteryl ester (CE) to transfer from HDL to TRL, thereby increasing the cholesterol content of remnant particles and lowering HDL-C. Metabolic studies have established that VLDL- and chylomicron- remnants are cleared from the circulation by the LDL receptor and possibly other receptors binding to apoB on the particle surface and facilitating endocytosis and degradation. ^2, 20^ Cluster 1 SNPs which reduce the activity of LDL receptor or alter the ligand apoB affect remnant clearance and cause increases in the concentration of both TRL/remnant and LDL particles with a consequent substantial rise in plasma apoB.

This examination of the relationship of TRL/remnant lipoproteins and LDL with risk of an ASCVD event in the UK Biobank population led to two major conclusions. First, it mattered which biomarker was utilised as an index of the abundance of TRL remnants – plasma TG, TRL/remnant-C, and VLDL-C all gave different answers – and second, regardless of the approach used, it appeared that ASCVD risk per unit change in TRL/remnant concentration was substantially higher than that seen for LDL. Further, we identified two distinct clusters of SNPs that affected TRL/remnant-C. In cluster 1 which included SNPs linked to genes known to alter the activity of lipoprotein receptor pathways (such as *PCSK9*, *APOB*, *LDLR*) change in TRL/remnant-C was accompanied by substantial changes in LDL-C and plasma apoB levels. The effect of cluster 1 SNPs on TG was modest, possibly because the associated genes do not impact on the levels of TG-rich, newly secreted TRL particles entering the circulation. In cluster 2 which included SNPs in genes linked to variation in TRL lipolysis (such as *LPL*, *APOA5*, *APOC3*) changes in TRL/remnant-C of a similar magnitude to those seen in cluster 1 were accompanied by much smaller changes in LDL-C and apoB. These findings may be interpreted in light of the metabolism of apoB-containing lipoproteins as summarised in Figure 6. That is, SNPs influencing the efficiency of receptor pathways (the LDL receptor *per se* or the ligand apoB) will likely alter, in concert, remnant lipoprotein and LDL clearance^20^ while SNPs affecting the lipolysis pathway will influence the rate of remnant formation.^2, 20^ The observations that the gradient of ASCVD risk per unit increment in apoB was higher in cluster 2 compared to cluster 1, and that the odds ratio for ASCVD risk for TRL/remnant-C was greater than for LDL-C in multivariable models of the whole cohort, are compatible with the concept that TRL/remnant particles have a higher atherogenic potential than LDL.

In the main, there are two complementary approaches to conducting Mendelian randomisation analyses, one involves the development of instrumental variables based on SNPs in a single gene or a few genes thought to alter specifically the risk factor (exposure) of interest, the other is a more agnostic, polygenic approach where all informative SNPs identified by a GWAS of the risk factor are included in the assessment.^21^ For reasons described below, we adopted the latter method in the present study and to our knowledge this is the first report of a GWAS using specifically the cholesterol content of TRL/remnants as the exposure of interest. An example of the first approach is a large, combined cohort analysis^8^ which showed that SNPs linked to the lipoprotein lipase (*LPL*) gene with effects on TG, and SNPs linked to the LDL receptor gene (*LDLR*) with effects on LDL-C had the same impact on ASCVD risk when the associated change in apoB was equalised. Further, it was found that genetic variants linked to plasma TG had no predictive value in models that included apoB. These findings led to the conclusion that the primary biomarker of risk was the number of apoB-containing particles, be they TRL or LDL, and that each particle had a similar atherogenic potential. This was the interpretation also of a recent study that found an apparently flat association of the TG to LDL-C ratio with risk once apoB was standardised.^10, 22^ In contrast, extensive reports from the Copenhagen General Population Study^1, 11–13, 23,21^ and other cohorts^12^ indicated that TRL cholesterol or remnant cholesterol was associated with a higher CHD risk per mmol/l increase than LDL-C, and since remnants have a higher cholesterol/apoB ratio than LDL^2^ this implies a greater per-particle atherogenicity for the former compared to the latter.

The results of the present investigation based on the UK biobank are in accord with these findings in the Danish population; that is, we found in Mendelian randomisation models using a range of SNP sets that TRL/remnant-C gave a higher relative risk ratio for ASCVD outcome than LDL-C per unit change in cholesterol, and that TG, VLDL-C, and TRL/remnant-C retained a significant association with risk when apoB was included in the multivariable model. The discordancy between the present and earlier^8^ results regarding the risk associated with apoB in TRL/remnants versus LDL may be attributable, at least in part, to the choice of genetic instrumental variables and plasma lipid exposures. In adopting a polygenic approach to Mendelian randomisation, our aim was to reflect better the complexity of metabolic pathways that determine TRL/remnant concentrations; TRL/remnant levels are the net result of multiple factors regulating the rates of formation and removal of these particles of which the action of lipoprotein lipase is just one (important) element.^20^ Further, use of variants linked to the LDL receptor gene as an instrumental variable for LDL-C is compromised by the revelation from metabolic studies that the LDL receptor is involved also in TRL/remnant clearance^2, 20^ – an observation that explains the increase in remnants in familial hypercholesterolemia and the reduction in TRL/remnant particle concentration on statin treatment.^20, 24, 25^ We found that SNPs affecting the receptor pathway (cluster 1 which includes SNPs in the LDL receptor gene) had little effect on plasma TG as an exposure (confirming earlier findings^8^), but here show that they do indeed influence TRL/remnant-C. From a metabolic perspective, given the inter-relationships between lipoproteins in the VLDL-remnant-LDL pathway, it may be difficult to identify instrumental variables based on single genes that permit a clear distinction between lipoprotein fractions in their contribution to ASCVD risk.

The main drawback in the use of a polygenic approach to Mendelian randomisation analysis is that it is difficult to account fully for potential SNP pleiotropic effects that may confound the relationship of the exposure (apoB) to outcome (ASCVD event) and an unknown number of variants may be invalid as instrumental variables.^18, 19, 21^ Although the SNP set we used was selected on the basis of the association with TRL/remnant-C and LDL-C, some variants may exhibit pleiotropy influencing ASCVD positively or negatively out with the effect on apoB.^18^ However, close agreement in the results from the range of SNP sets examined, and from the different statistical methods of conducting Mendelian randomisation analyses that accommodate potential pleiotropic effects, supports the validity of our interpretation.^21^

It is worthwhile noting that the two SNP clusters were defined empirically based on the observed effect size for apoB relative to TRL/remnant-C rather than any preconception as to which SNPs/genes should be grouped together based on their role in lipid metabolism or their relationship to ASCVD outcome. Since the proportion of TRL/remnant particles to LDL was higher in the ‘lipolysis’ cluster 2 compared to the ‘receptor’ cluster 1 and each particle type carries one apoB moiety, we were able to gain insight into the quantitative association of apoB with ASCVD risk in TRL/remnants versus LDL. Our observations are compatible with, and help explain, earlier findings that subjects with triglyceride-lowering variants (such as those causing angiopoietin-like proteins 3 and 4 loss-of-function) had a substantial reduction in ASCVD risk beyond that attributable to decreased LDL-C.^26^

We revisited the observation^10^ that if apoB is maintained constant in a prediction model then the ratio of TG to LDL-C (as an index of remnant versus LDL particles) shows no association with risk, since it was based in part on the same (UK biobank) data, and appeared to be at odds with the findings of the present study. It was found that the nature of the relationship of the TG or TRL/remnant-C to LDL-C ratio with ASCVD risk, corrected for apoB, differed depending on whether HDL-C was included in the model. When HDL-C was not in the model, both ratios show a significant association with ASCVD risk in keeping with our genetic analyses. Arguably, inclusion of HDL-C in prediction models can be regarded as at least a partial overcorrection since the same metabolic process that generates remnants also depletes the HDL fraction of cholesterol (Figure 6).^27, 28, 1, 2, 11, 29^

The key question that arises from the present and earlier investigations is what is the basis of a remnant particle’s enhanced atherogenicity?^29, 30^ Remnant particles contain more cholesterol per apoB and so if the ‘atherogenicity’ of TRL/remnant-C is higher than LDL-C then this implies that the per-particle (per apoB) impact on atherosclerotic processes must be even greater. This could be attributed to apoproteins present on the remnant’s surface that enhance interaction with proteoglycans (apoE, apoC-III),^30^ or the presence of lysophospholipids, partially digested glycerides or minor lipids such as ceramide that are cytotoxic or stimulate inflammatory mechanisms in the artery wall. Observations such as those reported here prompt further evaluation of the role of TRL/remnant particles in atherogenesis and the promotion of plaque instability.

The present investigation has limitations. The main one is that the analysis was performed using a single, large cohort of white European ancestry. Thus, there is a need to replicate the findings in other populations where appropriate measurements are available. However, the broad agreement between the present results and those from the Copenhagen General Population Study^1, 11–13, 23^ do support their generalisability. Our ‘measurement’ of TRL/remnant-C was indirect and there will be accumulated analytical errors in the values obtained. No measurement of TRL/remnant apoB was available and so the association of apoB with risk in TRL/remnants versus LDL was inferred by looking at clusters of SNPs that differentially affected the levels of TRL/remnants versus LDL. Finally, in using a polygenic approach, we cannot eliminate the possibility that pleiotropic effects confounded the results, although the likelihood that this was a major issue is diminished in light of the multiple statistical methods used to validate the results of the Mendelian randomisation analyses.

In conclusion, we have shown that choice of lipoprotein biomarker (exposure) to reflect the abundance of TRL/remnants has a substantial impact on the perceived quantitative association of genetic variants with ASCVD risk. The SNP cluster analysis indicated that association of apoB with ASCVD risk is not uniform, rather it depends on which particle the apoB resides; TRL/remnant particles appear to have an inherent atherogenicity that is greater than that of LDL. The implication of the present and earlier findings^4, 26^ is that interventions to regulate TRL/remnant lipoproteins may prove disproportionately beneficial. There is also the need to develop better ways of assessing remnant concentrations and to improve understanding the molecular basis of the atherogenicity of this lipoprotein species.

## Supplementary Material

Supplementary Material

## Figures and Tables

**Table 1:** Multivariable Mendelian randomisation analyses ^a^ of combined association of LDL-C and TRL/remnant-C with risk of an ASCVD event.

SNP set and exposure	Nr of SNPs	ASCVD causal effect estimate (OR per unit change [95% CI])^b^	ASCVD causal effect estimate (OR per SD change [95% CI])^c^	P-value
**Targeted gene analysis**	n=61			
*Univariable*				
LDL-C		1.49 [1.39, 1.59]	1.38 [1.31, 1.46]	3.8x10^-32^
*Multivariable*				
LDL-C		1.28 [1.14, 1.42]	1.22 [1.11, 1.34]	1.7x10^-05^
TRL/remnant-C		2.28 [1.39, 3.74]	1.28 [1.11, 1.49]	0.001
**Previously published SNP set**	n=178			
LDL-C		1.27 [1.13, 1.41]	1.21 [1.11, 1.33]	2.8x10^-05^
TRL/remnant-C		2.05 [1.34, 3.12]	1.24 [1.09, 1.41]	0.00084
**GWAS: Tier 1**	n=380			
LDL-C		1.28 [1.18, 1.39]	1.22 [1.15, 1.31]	6.4x10^-10^
TRL/remnant-C		1.87 [1.39, 2.50]	1.21 [1.11, 1.32]	3.0x10^-05^
**GWAS: Tier 2**	n=752			
LDL-C		1.24 [1.15, 1.34]	1.19 [1.12, 1.27]	9.3x10^-08^
TRL/remnant-C		1.94 [1.46, 2.58]	1.22 [1.12, 1.33]	5.9x10^-06^
**GWAS Tier 3**	n=1222			
LDL-C		1.24 [1.15, 1.32]	1.19 [1.12, 1.26]	3.6x10^-09^
TRL/remnant-C		1.93 [1.51, 2.47]	1.22 [1.13, 1.31	1.8x10^-07^

a Multivariable Mendelian randomisation models used the inverse-variance weighted (IVW) method. Potential impact of SNP pleiotropic effects was tested in Supplementary Table 3. For the targeted gene analysis only, the odds ratio for LDL-C in a univariable model is compared to that for LDL-C when it is included in a multivariable model with TRL/remnant-C.

b Odds Ratio per 1.0 mmol/L genetically-determined change in LDL-C and TRL/remnant-C.

c Odds Ratio per genetically-determined population standard deviation (SD) for LDL-C and TRL/remnant-C (TRL/remnant-C SD = 0.30 mmol/L, LDL-C SD = 0.82 mmol/L).

**Table 2:** Multivariable Mendelian randomisation models^a^ of apoB plus lipid variables and risk of an ASCVD event.

Multivariable MR model	Nr of SNPs	ASCVD causal effect estimate (OR per unit change [95% CI])^b^	ASCVD causal effect estimate (OR per SD change [95% CI])^c^	P-value
**Model 1**	n=178			
ApoB		3.03 [2.44, 3.78]	1.29 [1.23, 1.36]	3.1x 10^-23^
TG		1.17 [1.07, 1.28]	1.17 [1.07, 1.28]	0.00074
**Model 2**	n=1222			
ApoB		2.93 [2.54, 3.38]	1.28 [1.24, 1.33]	1.2x10^-48^
TG		1.15 [1.09, 1.21]	1.15 [1.09, 1.21]	2.2x10^-07^
**Model 3**	n=1222			
ApoB		2.16 [1.71, 2.71]	1.19 [1.13, 1.26]	6x10^-11^
TRL/remnant-C		1.82 [1.42, 2.32]	1.20 [1.11, 1.29]	1.8x10^-06^
**Model 4**	n=1222			
ApoB		2.70 [2.30, 3.17]	1.26 [1.21, 1.31]	2.4x10^-34^
VLDL-C		1.40 [1.23, 1.60]	1.16 [1.10, 1.23]	2.8x10^-07^

a Multi-variable randomisation models used the inverse-variance weighted method. The previously published SNP set and Tier 3 SNP set were used. Potential impact of SNP pleiotropic effects was tested as set out in Supplementary Table 3.

b Odds Ratio per 1.0 g/L change in apoB or per 1.0 mmol/L change in LDL-C and TRL/remnant-C

c Odds Ratio per population standard deviation (SD) change in respective variable (TG SD = 1.0 mmol/L, apoB SD = 0.23 g/L, TRL/remnant-C SD = 0.30 mmol/L, VLDL-C SD = 0.43 mmol/L)

## Data Availability

Data analyzed in this study was made available through the UK Biobank. Data is available up on application to UK Biobank https://www.ukbiobank.ac.uk.
