# Supplementary material for: Triglyceride-rich lipoprotein remnants, low-density lipoproteins, and their relative contribution to risk of atherosclerotic cardiovascular disease in the UK Biobank population
Source: Eur Heart J. Author manuscript; Available in PMC 2023 Oct 15. (PMC10576615; doi:10.1093/eurheartj/ehad337)
Supplement: Supplementary Material [file EMS180732-supplement-Supplementary_Material.pdf]

SUPPLEMENTAL FILE

Triglyceride-rich lipoprotein remnants, low-density lipoproteins, and risk of coronary heart disease: a UK Biobank study

Elias Björnson[1], Martin Adiels[1,2], Marja-Riitta Taskinen[3], Stephen Burgess[4,5], Aidin Rawshani[1], Jan Borén[1] and Chris J Packard[6]

[1]Department of Molecular and Clinical Medicine, University of Gothenburg, Gothenburg, Sweden; [2]School of Public Health and Community Medicine, Institute of Medicine, University of Gothenburg, Gothenburg, Sweden. [3]Research Program for Clinical and Molecular Metabolism, University of Helsinki; University of Helsinki, Finland; [4]MRC Biostatistics Unit, University of Cambridge, Cambridge, UK, [5]Cardiovascular Epidemiology Unit, Department of Public Health and Primary Care, University of Cambridge, Cambridge, UK, [6]Institute of Cardiovascular and Medical Sciences, University of Glasgow, Glasgow, UK.

Supplementary Table 1. 20 SNPs in clusters 1 and 2 with largest effect sizes (effects are minor/major allele). *P-values reported as zero if lower than the level of machine precision (2.225E-308)

A. SNPs in cluster 1 with largest effect sizes (n=20).

Gene	rs-number	Beta coefficient ApoB	Beta coefficient LDL-C	Beta coefficient TRL/rem-C	P-value* ApoB	P-value* LDL-C	P-value* TRL/rem-C	TRL/rem-C to apoB beta ratio	TRL/rem-C to LDL-C beta ratio
PCSK9	rs11591147	-0,0972	-0,371	-0,0665	0	0	2,45E-143	0,68	0,18
TOMM40	rs11668327	-0,0508	-0,153	-0,0273	0	0	1,49E-182	0,54	0,18
CEACAM16_BCL3	rs11881756	-0,0409	-0,121	-0,0165	0	0	2,21E-53	0,4	0,14
APOC2_APOC4	rs12721109	-0,117	-0,347	-0,0456	0	0	6,82E-98	0,39	0,13
PVRL2	rs142042446	0,0273	0,0797	0,0142	0	0	2,34E-80	0,52	0,18
BCAM	rs28399637	-0,0228	-0,0698	-0,0128	0	1,45E-268	1,78E-65	0,56	0,18
PVRL2	rs387976	0,0384	0,12	0,0369	0	6,26E-252	3,25E-169	0,96	0,31
APOC1P1_APOC1	rs60049679	-0,0734	-0,276	-0,0458	1,57E-221	2,35E-249	4,38E-50	0,62	0,17
LDLR	rs72658867	0,0516	0,146	0,032	0	9,08E-217	6,15E-75	0,62	0,22
TOMM40	rs115881343	-0,0266	-0,0833	-0,0142	1,96E-268	2,27E-210	1,7E-44	0,53	0,17
PVRL2	rs41289512	0,0141	0,054	0,0093	1,12E-162	6,01E-190	5,39E-41	0,66	0,17
APOB_LOC645949	rs10166144	-0,0163	-0,0607	-0,0111	1,98E-165	1,13E-183	7,4E-45	0,68	0,18
LDLR	rs6511721	-0,0175	-0,0505	-0,0077	1,76E-236	4,38E-158	2,76E-27	0,44	0,15
SMARCA4	rs12052058	-0,042	-0,11	-0,032	1,38E-260	2,31E-144	1,2E-85	0,76	0,29
CEACAM16_BCL3	rs2965109	-0,031	-0,124	-0,0198	1,85E-111	2,18E-141	7,65E-27	0,64	0,16
APOC1	rs12691088	-0,0264	-0,08	-0,0125	4,71E-177	9,88E-130	1,22E-23	0,47	0,16
KANK2	rs7188	0,015	0,0604	0,0112	4,91E-100	1,85E-129	1,95E-32	0,75	0,19
APOB_C2orf43	rs6547409	-0,0118	-0,046	-0,00715	7,33E-107	4,43E-129	2,4E-23	0,61	0,16
LDLR	rs73015030	-0,0353	-0,107	-0,02	7,73E-163	3,24E-119	7,62E-31	0,57	0,19
MYBPHL	rs55660224	-0,0139	-0,052	-0,00902	3,86E-98	4,57E-109	2,18E-24	0,65	0,17

B. SNPs in cluster 2 with largest effect sizes (n=20).

Gene	rs-number	Beta coefficient ApoB	Beta coefficient LDL-C	Beta coefficient TRL/rem-C	P-value* ApoB	P-value* LDL-C	P-value* TRL/rem-C	TRL/rem-C to apoB beta ratio	TRL/rem-C to LDL-C beta ratio
BUD13_LINC00900	rs12280753	0,0214	0,0651	0,0531	8,13E-105	3,31E-78	0	2,48	0,82
GCKR	rs1260326	0,0148	0,0419	0,026	9,75E-172	2,97E-110	8,93E-294	1,76	0,62
LPL	rs295	-0,00932	-0,0203	-0,0267	5,49E-54	1,61E-21	1E-241	2,86	1,3
APOA4_APOC3	rs10790164	0,0198	0,0632	0,0555	4,86E-43	1,32E-35	8,03E-185	2,8	0,88
BUD13_LINC00900	rs506222	0,0119	0,0368	0,0308	2,43E-43	2,36E-33	9,52E-157	2,59	0,84
NCAN	rs2228603	-0,0243	-0,099	-0,0344	9,8E-135	1,68E-177	4,58E-151	1,42	0,35
LPL	rs268	0,0239	0,04	0,0554	1,2E-33	1,12E-08	2,3E-97	2,32	1,4
LPL_SLC18A1	rs11204087	-0,00464	-0,00995	-0,0137	5,17E-19	0,00000007	1,65E-86	2,95	1,4
VPS37D_MLXIPL	rs13230514	-0,00489	-0,00638	-0,0146	8,78E-18	0,00154	3,53E-82	2,99	2,3
ANGPTL4	rs116843064	-0,011	-0,0243	-0,0466	4,91E-09	0,000273	2,28E-76	4,24	1,9
LPL_SLC18A1	rs10103634	-0,0031	-0,00578	-0,0133	1,78E-08	0,00302	1,3E-73	4,29	2,3
BUD13_LINC00900	rs879858	0,0167	0,0557	0,048	4,92E-16	2,03E-14	5,85E-68	2,87	0,86
VEGFA_LOC100132354	rs4711750	0,00592	0,015	0,0116	2,08E-30	2,55E-16	1,23E-63	1,96	0,77
TRIB1_LINC00861	rs7832357	0,00672	0,0221	0,0118	7,61E-35	2,08E-30	6,13E-59	1,76	0,53
BUD13_LINC00900	rs180358	-0,004	-0,0117	-0,0131	2,22E-10	1,64E-07	1,3E-54	3,28	1,1
ZNF512	rs1881396	-0,0075	-0,0212	-0,0128	2,74E-33	7,81E-22	1,27E-53	1,71	0,6
MAU2	rs3764567	-0,00663	-0,0278	-0,0103	1,11E-34	3,82E-48	2,46E-46	1,55	0,37
HLA-DQB1_HLA-DQA2	rs17405319	0,00896	0,0305	0,0126	1,57E-40	1,56E-37	1,34E-44	1,41	0,41
TRIB1	rs74737417	-0,00642	-0,016	-0,02	2,23E-09	0,0000242	3,95E-44	3,12	1,2
LPL_SLC18A1	rs1441778	-0,00375	-0,00724	-0,0128	1,05E-07	0,00372	2,92E-42	3,41	1,8

Supplementary Table 2.

Definition of CHD outcomes

**CHD outcome**		**Individuals, n=487,202**
Non-fatal myocardial infarction (MI)	ICD 9 codes 410, 4110, 412, 42979<br>ICD 10 codes I21, I22, I23, I241, I252	Prevalent events n = 6,577<br>Incident events n = 17,356
Fatal MI	ICD 10 codes I21, I23, I241, I251, 1252, I253, I255-I259	Incident events n = 3,850
Coronary revascularisation	**Operational procedures**<br>Codes K501, K40-K44	Prevalent events n = 2,845<br>Incident events n = 3,571
Unique CHD outcomes	First event of above	Prevalent events n = 8,391<br>Incident events n = 20,792<br>Total events = 29,183

Incident events based on approximately 12 years of follow (as of January 2021).

Supplementary Table 3 A B – Replication analysis

Replication of key findings in the CARDIoGRAMplusC4D data set.

Of the 1125 SNPs identified as associated with TRL/remnant-C (TRL/rem-C) and/or LDL-C in the UK Biobank (Supplementary Figure 1), 1049 were present also in the CARDIoGRAMplusC4D SNP set. The beta coefficients for lipid traits estimated using the UK Biobank were applied to SNP-outcome data in CARDIoGRAMplusC4D (myocardial infarction) using two-sample multivariable MR models.

A further analysis in the CARDIoGRAMplusC4D data set was done using univariable models examining the association of apoB with CHD within SNP clusters 1 and 2.

A. Two-sample multivariable models

	Nr of SNPS	CHD causal effect estimate (OR per unit change [95% CI])	CHD causal effect estimate (OR per SD change [95% CI])	P-value
**Model with apoB + TRL-C**	1049			
ApoB		2.90 [2.14, 3.93]	1.28 [1.19, 1.37]	5.6×10^{-12}
TRL/remnant-C		2.01 [1.48, 2.72]	1.25 [1.13, 1.38]	8.3×10^{-06}
**Model with LDL-C + TRL-C**	1049			
LDL-C		1.42 [1.30, 1.56]	1.33 [1.24, 1.44]	9.6×10^{-15}
TRL/remnant-C		1.84 [1.36, 2.49]	1.22 [1.11, 1.34]	6.8×10^{-05}

B. Two-sample univariable models

	Nr of SNPS	CHD causal effect estimate (OR per unit change [95% CI])	CHD causal effect estimate (OR per SD change [95% CI])	P-value
**Cluster 1 SNPs**	509			
ApoB		4.78 [3.77, 6.07]	1.44 [1.36, 1.52]	7.6×10^{-38}
**Cluster 2 SNPs**	388			
ApoB		9.38 [6.13, 14.34]	1.68 [1.52, 1.85]	5.5×10^{-25}

Conclusion

Key results from the UK Biobank analyses relating lipoprotein variables to CHD outcome were largely replicated in the CARDIoGRAMplus C4D data set. That is, TG, TRL/remnant-C and VLDL-C were indepedenet predictors of CHD risk in models including apoB, and the gradient of association of apoB with CHD risk for cluster 2 was greater than for cluster 1.

Sensitivity analyses

A number of sensitivity analyses were undertaken to determine the influence of:

(1) *Choice of threshold for linkage disequilibrium* – further pruning was performed using $r^2<0.01$ and $r^2<0.001$. Results are given in **Supplementary Table 4**.

(2) *Pleiotropic effects* - Mendelian randomisation model results presented in the main text are derived using the inverse-variance weighted (IVW) method. Other methods are better equipped to handle possible bias stemming from pleiotropic effects and/or effects from inclusion of invalid instrumental variables. Results from these additional methods (ROBUST-IVW, LASSO and the MR-EGGER) are given in **Supplementary Table 5**.

(3) *Weak instrumental bias* - Replication of results in a separate data set was used to check possible influence of weak instrumental bias (since exposure-outcome associations were tested in the same data set that was used to derive SNP exposure estimates). A replication analysis was undertaken by applying the beta coefficients for lipid traits in the UK Biobank SNP set to the CARDIoGRAMplusC4D outcome data. Results are reported above in **Supplementary Table 3**.

Supplementary Table 4

Impact of choice of linkage disequilibrium (LD) significance threshold (r^2<0.1, r^2<0.01, r^2<0.001) for pruning of GWAS SNP set.

Table 4 Influence of LD threshold on multivariate models of TRL/remnant-C and LDL-C

Exposure	Nr SNPs	CHD causal effect estimate (OR per unit change [95% CI])[b]	CHD causal effect estimate (OR per SD change [95% CI])[c]	P value	P-diff
**r^2<0.1**	1125				
LDL-C		1.37 [1.27, 1.48]	1.29 [1.22, 1.38]	4.0×10^{-16}	
TRL/remnant-C		2.59 [1.99, 3.36]	1.36 [1.25, 1.48]	1.2×10^{-12}	$1.4\text{x}10^{-4}$
**r^2<0.01**	663				
LDL-C		1.35 [1.19, 1.54]	1.28 [1.15, 1.42]	3.6×10^{-06}	
TRL/remnant-C		2.90 [1.91, 4.40]	1.41 [1.23, 1.61]	5.2×10^{-07}	$4.6\text{x}10^{-3}$
**r^2<0.001**	469				
LDL-C		1.32 [1.14, 1.55]	1.26 [1.11, 1.43]	3.6×10^{-04}	
TRL/remnant-C		2.89 [1.74, 4.82]	1.41 [1.20, 1.66]	4.4×10^{-05}	0.017

1. Multivariate models including LDL-C and TRL/remnant-C as exposures were constructed using SNP sets derived as in Supplementary Figure 1 using significance thresholds of varying stringency to prune SNPs in linkage disequilibrium.
2. Odds ratios and confidence limits are given for each model as is the P value for association with CHD outcomes for each exposure and a P value for difference between the two odds ratios (determined using the function *linearHypothesis* in the R-package *car*)

Conclusions

1. Relative risk estimates for CHD were in close agreement across all significance thresholds for r^2.
2. Use of more stringent r^2 thresholds (<0.01, <0.001) reduced the number of SNPs and the statistical power to detect differences in the association of exposure with CHD risk.
3. The r^2 threshold of <0.1 as adopted in the main analysis gave maximum power and generated estimated relative risk ratios in close agreement with more stringent thresholds.

Supplementary Table 5

BACKGROUND

The inverse-variance weighted method for Mendelian randomisation analysis assumes all SNP variants are 'valid instrumental variables', that is the effect on outcome (CHD event) is only through the effect on the designated exposure (risk factor). This may be a valid assumption when SNPs/genes are known to be involved in lipid metabolism. Use of larger, polygenic SNP sets identified by GWAS may include variants that do not meet this assumption i.e. are 'invalid'. The requirement then is to assess statistically the number of possible invalid variants and their potential to bias the calculated odds ratios. Several statistical methodologies have been devised to test the possibility that SNP horizontal pleiotropy may have biased the association of the exposure with outcome.

Supplementary Table 5 presents an assessment of potential pleiotropic effects. We used the multivariable model of TRL/remnant-C plus LDL-C as a test of pleiotropy in the GWAS-derived SNP set. The model was replicated using in addition to standard MR-IVW, the outlier-tolerant ROBUST weighted-IVW,[1] the Lasso,[2] and the Egger statistical methods which evaluate, and are tolerant of, outliers and pleiotropic effects respectively.

Multivariable Mendelian randomisation (MR) analysis comparing inverse-variance weighted (IVW) method, ROBUST weighted-IVW method, Lasso method and Egger method.

	Method	Nr of SNPS	Valid SNPs	CHD causal effect estimate (OR per unit change [95% CI])	CHD causal effect estimate (OR per SD change [95% CI])	P-value
**Multivariable**		**1125**				
LDL-C	IVW		1125	1.37 [1.27, 1.48]	1.29 [1.22, 1.38]	4.0×10^{-16}
TRL/remnant-C	IVW		1125	2.59 [1.99, 3.36]	1.36 [1.25, 1.48]	1.2×10^{-12}
**Multivariable**		**1125**				
LDL-C	ROBUST-IVW		1125	1.42 [1.30, 1.54]	1.33 [1.24, 1.43]	3.4×10^{-16}
TRL/remnant-C	ROBUST-IVW		1125	2.31 [1.73, 3.07]	1.31 [1.19, 1.44]	1.1×10^{-8}
**Multivariable**		**1125**				
LDL-C	Lasso		972	1.43 [1.35, 1.52]	1.34 [1.28, 1.41]	3.3×10^{-32}
TRL/remnant-C	Lasso		972	2.18 [1.77, 2.69]	1.29 [1.20, 1.38]	1.6×10^{-13}
**Multivariable**		**1125**				
LDL-C	Egger		1125	1.35 [1.25, 1.46]	1.28 [1.20, 1.37]	3.1×10^{-13}
TRL/remnant-C	Egger		1125	2.45 [1.84, 3.25]	1.34 [1.22, 1.46]	7.1×10^{-10}

CONCLUSION

The close agreement of causal estimates across the four statistical approaches provides a high degree of confidence in the application of the inverse-variance weighted method in multivariable Mendelian randomisation models used in the present analysis.

Supplementary Figure 1. Flowchart of subject- and SNP selection as basis for the Mendelian Randomisation analyses.

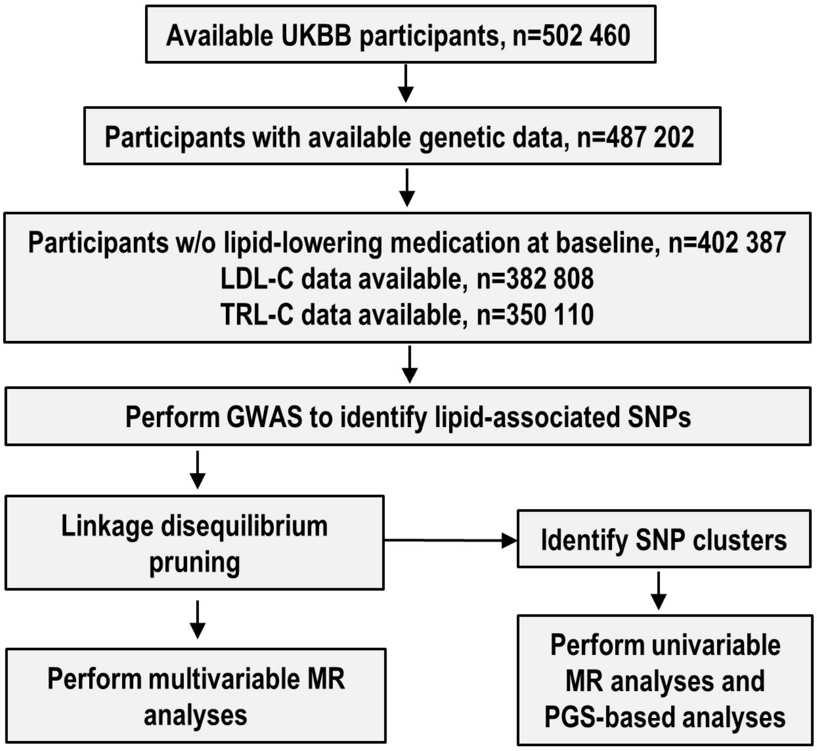

1. Genetic instruments (SNPs) and beta coefficients were determined in subjects not on lipid-lowering therapy in whom appropriate lipoprotein levels were available (in the case of TRL/remnant-C the number of subjects was 350,110). This selection criterion eliminated the confounding effects of drugs (mainly statins) on the SNP to exposure relationship.

2. The association of genetically-determined variation in lipoprotein variables with CHD outcome was explored using all subjects with appropriate genetic information available. The number of subjects in these analyses was 487,202 and included those off or on lipid-lowering treatment.

3. Analysis of the relationship between observed lipoprotein variables measured at baseline and incident CHD was undertaken in subjects free of CHD at baseline. The number of subjects meeting this criterion was 478,811.

Supplementary Figure 2

Association of indirectly measured TRL/remnant-C with TG and LDL-C in subjects not on lipid-lowering treatment.

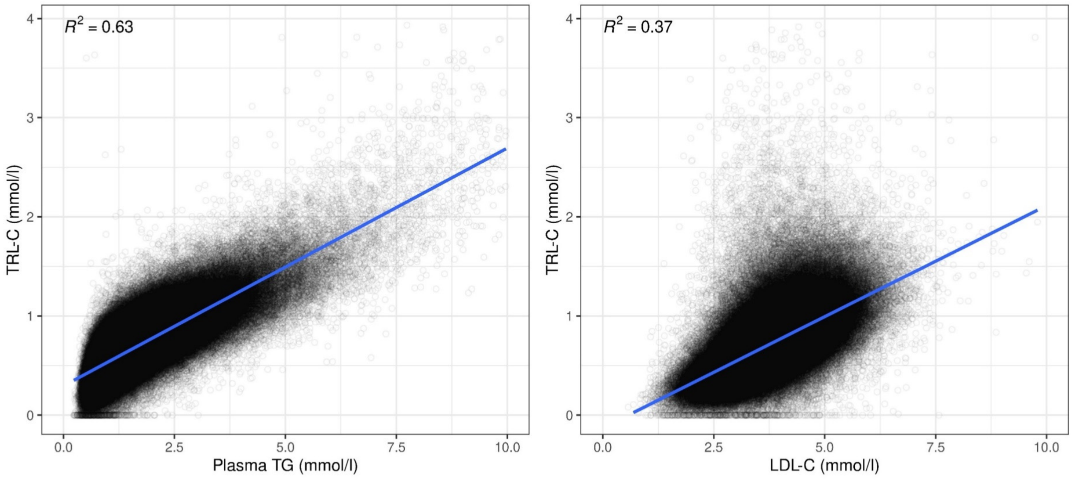

Supplementary Figure 3

Effect sizes of GWAS derived SNPs on apoB in relation to TG and LDL-C.

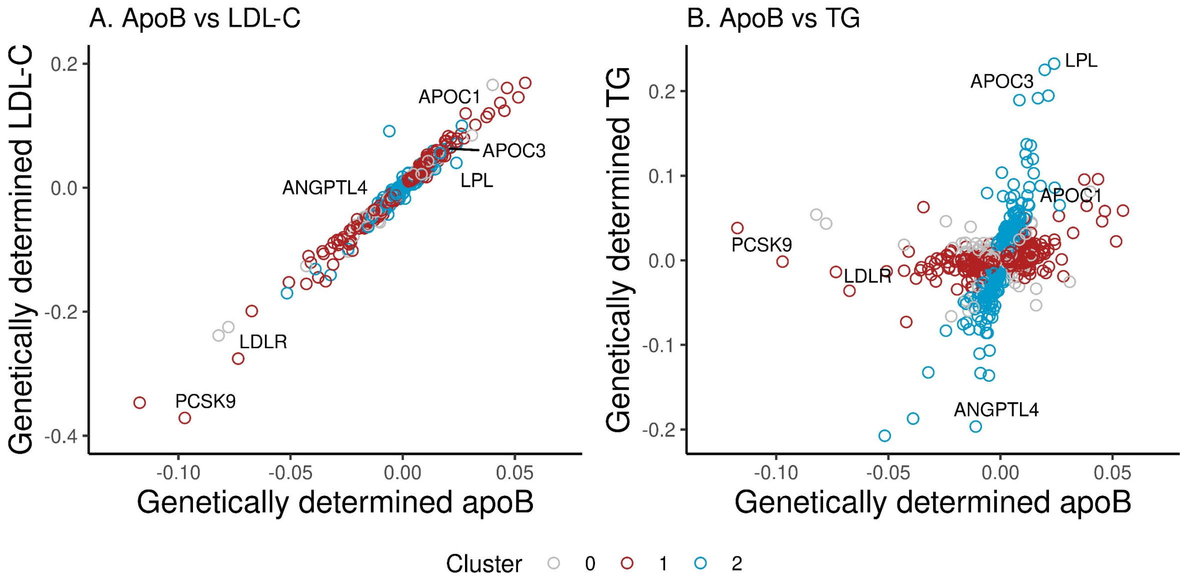

REFERENCES

1. Burgess S, Bowden J, Dudbridge F, Thompson SG. Robust instrumental variable methods using multiple candidate instruments with application to Mendelian randomization. arXiv preprint arXiv:1606.03729 2016.
2. Rees JMB, Wood AM, Dudbridge F, Burgess S. Robust methods in Mendelian randomization via penalization of heterogeneous causal estimates. PLoS One 2019;**14**(9):e0222362.
